# D-Aspartate Modulates Nociceptive-Specific Neuron Activity and Pain Threshold in Inflammatory and Neuropathic Pain Condition in Mice

**DOI:** 10.1155/2015/905906

**Published:** 2015-01-05

**Authors:** Serena Boccella, Valentina Vacca, Francesco Errico, Sara Marinelli, Marta Squillace, Francesca Guida, Anna Di Maio, Daniela Vitucci, Enza Palazzo, Vito De Novellis, Sabatino Maione, Flaminia Pavone, Alessandro Usiello

**Affiliations:** ^1^Pharmacology Division, Department of Experimental Medicine, The Second University of Naples, Via Costantinopoli 16, 80138 Naples, Italy; ^2^CNR, National Research Council, Cell Biology and Neurobiology Institute, Roma, Italy; ^3^IRCCS, Santa Lucia Foundation, Rome, Italy; ^4^Laboratory of Behavioural Neuroscience, Ceinge Biotecnologie Avanzate, Via G. Salvatore 486, 80145 Naples, Italy; ^5^Department of Molecular Medicine and Medical Biotechnology, University of Naples “Federico II”, Naples, Italy; ^6^Department of Anaesthesiology, Surgery and Emergency, The Second University of Naples, Naples, Italy; ^7^Department of Environmental, Biological and Pharmaceutical Sciences and Technologies, The Second University of Naples, Naples, Italy

## Abstract

D-Aspartate (D-Asp) is a free D-amino acid found in the mammalian brain with a temporal-dependent concentration based on the postnatal expression of its metabolizing enzyme D-aspartate oxidase (DDO). D-Asp acts as an agonist on NMDA receptors (NMDARs). Accordingly, high levels of D-Asp in knockout mice for *Ddo* gene (*Ddo* ^−/−^) or in mice treated with D-Asp increase NMDAR-dependent processes. We have here evaluated in *Ddo* ^−/−^ mice the effect of high levels of free D-Asp on the long-term plastic changes along the nociceptive pathway occurring in chronic and acute pain condition. We found that *Ddo* ^−/−^ mice show an increased evoked activity of the nociceptive specific (NS) neurons of the dorsal horn of the spinal cord (L4–L6) and a significant decrease of mechanical and thermal thresholds, as compared to control mice. Moreover, *Ddo* gene deletion exacerbated the nocifensive responses in the formalin test and slightly reduced pain thresholds in neuropathic mice up to 7 days after chronic constriction injury. These findings suggest that the NMDAR agonist, D-Asp, may play a role in the regulation of NS neuron electrophysiological activity and behavioral responses in physiological and pathological pain conditions.

## 1. Introduction

D-Aspartate (D-Asp) is abundantly found in the brain during embryonic and perinatal periods, while it strongly decreases during adulthood [1–3]. D-Asp is selectively metabolized by D-aspartate oxidase (DDO), the only enzyme that degrades bicarboxylic D-amino acids, including N-methyl-D-aspartate (NMDA) [4]. DDO is widely expressed in the adult brain, since its activity strongly increases from postnatal phase until 6 weeks of life [5]. In mammals, D-Asp plays a role in glutamatergic neurotransmission acting as an agonist on NMDA receptors (NMDARs) through the binding to each of the GluN2 subunits [6–9]. Accordingly,* in vitro* electrophysiological studies have demonstrated that D-Asp is able to induce inward currents in CA1 pyramidal neurons in an NMDAR-dependent manner [8]. Genetically engineered animal models with a deletion of the* Ddo* gene (*Ddo* ^−/−^), showing nonphysiological high levels of D-Asp, have been generated to gain insight into the physiological role of this D-amino acid and of its catabolic enzyme [10]. The levels of D-Asp were up to 10–20-fold higher in the brain of* Ddo* ^−/−^ mice, as compared to the wild type littermates [11]. In line with its pharmacological features, we have demonstrated that excessive levels of D-Asp in the brain, in both* Ddo* ^−/−^ mice and mice chronically treated with D-Asp, are associated with an enhanced NMDAR-dependent long term potentiation (LTP) in the hippocampus and a loss of the long term depression (LTD) in the striatum [6–9, 12]. Windup is the LTP analogous mechanism at spinal cord dorsal horn level, leading to central sensitization at the base of chronic pain development and establishment [13, 14]. Similar to LTP, windup relies on NMDAR activation-dependent mechanism. Indeed, intrathecal injection of NMDA yields exaggerated behavioral responses to thermal and low intensity mechanical stimuli [15] while the peripheral administration of MK801, a noncompetitive NMDAR antagonist, reduces the excitability of spinal cord neurons under chronic pain conditions and inhibits formalin-induced inflammatory pain [16].

In the present study,* Ddo* ^−/−^ mice were used to investigate the consequences of high D-Asp levels on pain responses and nociceptive specific (NS) neuron activity in chronic pain by analyzing (i) thermal and mechanical thresholds in naïve mice, (ii) the evoked activity of the nociceptive specific (NS) neurons of the dorsal horn of the spinal cord (L4–L6), (iii) mechanical allodynia in a model of neuropathic pain induced by the chronic constriction injury (CCI) of the sciatic nerve, and, finally, (iv) the nocifensive responses in the formalin test.

## 2. Methods

### 2.1. Animals

Knockout mice for the* Ddo* gene were generated as described previously [10]. Adult male and female wild type (*Ddo* ^+/+^) and knockout (*Ddo* ^−/−^) 90-day-old mice, derived from mating of heterozygous (*Ddo* ^+/−^) mice and back-crossed to the F5 generation to C57BL/6J strain, were used. Animals were housed in standard transparent plastic cages, in groups of 4, lined with sawdust under a standard 12/12-h light/dark cycle (07:00 AM/07:00 PM), with food and water available* ad libitum*. Testing was performed blind to treatment group to which each subject belonged. All procedures were in strict accordance with the Italian National law (DL116/92, application of the European Communities Council Directive 86/609/EEC) on care and handling of the animals and with the guidelines of the Committee for Research and Ethical Issues of IASP.

### 2.2. Surgery

*Ddo* ^−/−^ and wild type mice underwent the chronic constriction injury (CCI) of the sciatic nerve according to Bennett and Xie [17] for inducing neuropathic pain (day 0). Briefly, animals were anaesthetized with sodium pentobarbital (50 mg/kg, i.p.), the right sciatic nerve was exposed through a 1 cm longitudinal skin incision, and three ligatures were loosely tied around the nerve just proximal to the trifurcation. The wound was then closed with 4-0 silk suture. In the following, the injured right hind paw will be named as ipsilateral paw and the uninjured left hind paw will be named as contralateral paw. Control* Ddo* ^−/−^ and wild type mice underwent a sham surgery with exposure of the sciatic nerve without ligature.

### 2.3. Extracellular Recordings of Nociceptive Neurons

For* in vivo* single unit extracellular recording, experimental groups consisted of 6–8 mice which included at least 3/4 male and 3/4 female subjects with more than one neuron recorded in each mouse. The single extracellular recordings were performed 7 days after surgery in* Ddo* ^−/−^ and* Ddo* ^+/+^ female or male mice.

On the day of electrophysiological recording experiments (day 7), mice were initially anesthetized with sodium pentobarbital (50 mg/kg i.p.). After tracheal cannulation, a catheter was placed into the right external jugular vein to allow continuous infusion of propofol (5–10 mg/kg/h, i.v.). Spinal cord segments L4–L6 were exposed medially by laminectomy, near the dorsal root entry zone, up to a depth of 1 mm [18]. An elliptical rubber ring (about 3 × 5 mm) was tightly sealed with silicone gel onto the surface of the cord. This ring formed a trough with about 50 *μ*L capacity over the spinal segments used for topical spinal drug application. It also provided access to the spinal neurons that receive input from the ipsilateral paw, where the mechanical stimulation was applied. Animals were then secured in a stereotaxic apparatus (David Kopf Instruments, Tujunga, CA, USA) supported by clamps attached to the vertebral processes on either side of the exposure site. The exposed area of the spinal cord was initially framed by agar and then filled with mineral oil. Body temperature was maintained at 37°C with a temperature-controlled heating pad [19, 20]. A glass-insulated tungsten filament electrode (3–5 MΩ; FHC Frederick Haer & Co., ME, USA) was used to record single unit extracellular activity of dorsal horn NS neurons. NS neurons were defined as those neurons that respond only to high-intensity (noxious) stimulation [19]. To confirm NS response patterns, each neuron was characterized while applying a mechanical stimulation to the ipsilateral hind paw using a von Frey filament with 97.8 mN bending force (noxious stimulation) for 2 s until it buckled slightly [21]. Only neurons that responded specifically to the noxious hind paw stimulation, without responding to stimulation of the surrounding tissue, were included in sham and neuropathic mice recordings. The recorded signals were amplified and displayed on a digital storage oscilloscope to ensure that the unit under study was unambiguously discriminated throughout the experiment. Signals were also fed into a window discriminator, whose output was processed by an interface CED 1401 (Cambridge Electronic Design Ltd., UK) connected to a Pentium III PC. Spike2 software (CED, version 4) was used to create peristimulus rate histograms online and to store and analyze digital records of single unit activity offline. Configuration, shape, and height of the recorded action potentials were monitored and recorded continuously using a window discriminator and Spike2 software for online and offline analysis. This study only included neurons whose spike configuration remained constant and could be clearly discriminated from activity in the background throughout the experiment, indicating that the activity from one neuron only and from the same neuron was measured. The neuronal activity was expressed as spikes/sec (Hz). At the end of the experiment, each animal was killed with a lethal dose of urethane.

### 2.4. Behavioral Testing

Mechanical withdrawal threshold was tested by using a Dynamic Plantar Aesthesiometer (Ugo Basile, Model 37400), an apparatus that generates a mechanical force linearly increasing with time. The force is applied to the plantar surface of the mice hind paw, and the nociceptive threshold is defined as the force, in grams, at which the mouse withdraws its paw [22]. Neuropathic wild type and* Ddo* ^−/−^ mice were tested for the sensitivity of both ipsilateral and contralateral hind paws to normally nonnoxious punctuate mechanical stimuli, at different time intervals from postoperative day 3 to day 31 and reported in Figure 4. At each testing day, the ipsi- and contralateral withdrawal forces were taken as mean of three consecutive measurements per paw with 10 s interval between each measurement. Experimental data are expressed as mean ± S.E.M.

Thermal withdrawal threshold was measured by exposing the plantar surface of both hind paws to an infrared heat stimulus delivered through an automatic device (Plantar test, Ugo Basile, Comerio, Italy). This apparatus basically consists of a movable infrared generator placed below a glass pane upon which animal is placed. After an acclimation period, the infrared source placed under the glass floor was positioned directly beneath the hind paw and trial began. When the animal felt pain and withdrew its paw, the infrared source switched off and the reaction time counter stopped [23]. The withdrawal latency to the nearest 0.1 s was automatically determined. To avoid damage of hind paw skin tissue, a cut-off time of 15 s was imposed. Thermal withdrawal latency of both hind paws was recorded as the mean of the three trials (s) for three consecutive trials with at least 10 seconds between each trial and was plotted.

Formalin test was used to evaluate the response to inflammatory pain [24]. Formalin solution (20 *μ*L of 5% in saline) was subcutaneously (s.c.) injected into the dorsal surface of the right hind paw of mice using a microsyringe equipped with a 26-gauge needle. Mice were then put back into the chamber and the observation period started. The nocifensive responses, that is, the total amount of time the animal spent licking the injected paw, were taken as index of pain and measured in sec. Nocifensive responses were recorded continuously for 40 minutes and calculated in blocks of consecutive 5 min periods.

### 2.5. Data Analysis

For electrophysiology experiments 2-way ANOVA for repeated measures followed by Bonferroni post hoc test for multiple comparisons has been used to analyse statistical differences between the different groups of mice. Data derived from male and female mice were analyzed separately.

Concerning behavioral experiments data were expressed as mean ± standard error of the mean (S.E.M). Statistical analysis was carried out by using Student's *t*-test to analyse mechanical and thermal nociceptive threshold or two-way ANOVA for repeated measures and one-way ANOVA to evaluate mechanical allodynia and formalin-induced inflammatory pain. Data derived from male and female mice were analyzed separately as well as the two phases of formalin test considered.

## 3. Results

### 3.1. Effect of the* Ddo* Gene Ablation on NS Neuron Activity of Female Mice in Control and Neuropathic Pain Conditions

**Figure 1:**
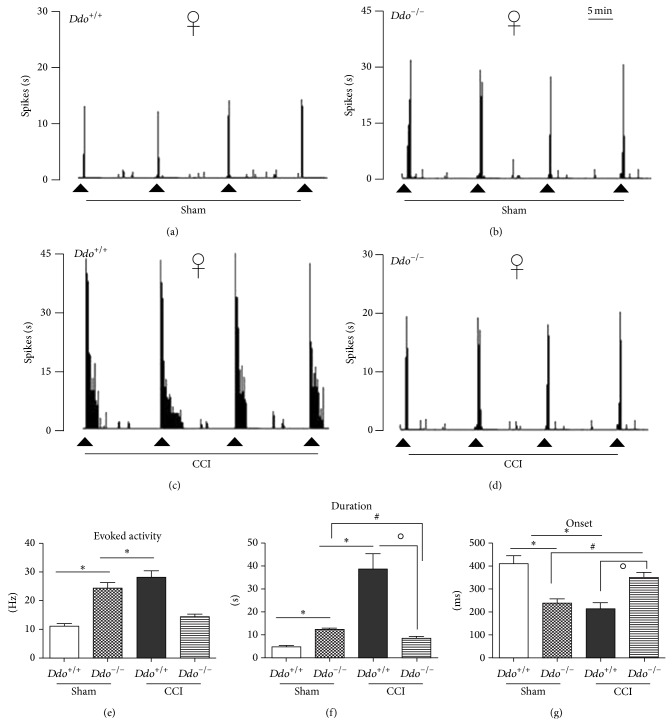
Representative ratemeters showing the responses of a single spinal NS neuron to a mechanical noxious stimulation (von Frey filaments 97.8 mN/2 sec) in* Ddo* ^+/+^ (a and c) and* Ddo* ^−/−^ (b and d) female mice 7 days after sham (a and b) or CCI (c and d) surgery. The lower panels show the evoked activity (e), the duration (f), and the onset (g) of the evoked activity induced by the noxious stimulation in NS neurons in sham and CCI* Ddo* ^+/+^ or* Ddo* ^−/−^ female mice. Small black arrows (a–d) indicate the noxious stimulation on mouse hind paw and grey scale bar indicates 5 min interval in ratemeter. Each point represents the mean ± S.E.M of 6–8 neurons of different groups of mice. *P* values <0.05 were considered statistically significant (two-way ANOVA followed by Bonferroni posttest, for comparisons between groups).  ^*^indicates significant differences versus sham/*Ddo* ^+/+^, ^#^significant differences versus sham/*Ddo* ^−/−^, and °significant differences versus CCI/*Ddo* ^+/+^.

The results are based on NS neurons (two cells recorded from each animal) at a depth of 0.7–1 mm from the surface of the spinal cord. This cell population was characterized by a mean rate of spontaneous firing of 0.015 ± 0.002 Hz. Thus, only cells showing this pattern of basal firing were chosen for the recordings. The electrophysiological studies have measured the onset of excitation (the time from the application of the stimulus artefact to the first evoked spike exceeding the average baseline value + 2 standard deviations), the frequency of the evoked excitatory responses, and the duration of excitation (the period in ms of the increased firing activity which exceeds the average baseline value + 2 standard deviations). The effect of* Ddo* gene deletion on NS neuron activity was firstly considered in female* Ddo* ^+/+^ and* Ddo* ^−/−^ mice. No change in the spontaneous firing activity of NS neurons has been found in the sham wild type mice (0.08 ± 0.001 spikes/s) (Figure 1) with respect to the naïve (not shown). In contrast, the deletion of the* Ddo* gene caused a significant decrease in the onset of the evoked activity and an increase in the duration and the frequency of the evoked activity (237 ± 18 ms, F_(3,10)_ = 21.02; 12 ± 0.66 s, F_(3,10)_ = 56.56; and 24.18 ± 2.11 Hz, F_(3,10)_ = 31, resp.; *P* < 0.05, *n* = 6) in sham* Ddo* ^−/−^ mice as compared to the sham wild type ones (Figure 1).

We observed an overall NS neuron hyperexcitability in CCI as compared with sham in wild type mice. In particular, we found a significant reduction in the onset and an increase in the frequency and in the duration of the evoked activity 7 days after CCI (202 ± 25.36 ms, F_(3,10)_ = 24.83; 27.8 ± 2.39 Hz, F_(3,10)_ = 42.10; and 38.5 ± 6.6 s, F_(3,10)_ = 25.69, resp.; *P* < 0.05, *n* = 6), as compared to the sham (410 ± 33.16 ms, 11 ± 1 Hz, 4.8 ± 0.8 s, resp.; *n* = 8) in wild type mice. Surprisingly, the CCI caused a significant increase in the onset and a reduction in the frequency and duration of the evoked activity (350 ± 20 ms, F_(3,10)_ = 17.64; 14.0 ± 1.28 Hz, F_(3,10)_ = 25.95; and 8.3 ± 1 s, F_(3,10)_ = 20.47, resp.; *P* < 0.05, *n* = 7) of NS neurons with respect to the shams in* Ddo* ^−/−^ mice (Figures 1(e), 1(f), and 1(g)). Representative ratemeter records show the activity of a single NS neuron in sham (Figures 1(a) and 1(b)) and in CCI (Figures 1(c) and 1(d)) wild type and* Ddo* ^−/−^mice.

### 3.2. Effect of the* Ddo* Gene Ablation on NS Neuron Activity of Male Mice in Control and Neuropathic Pain Conditions

**Figure 2:**
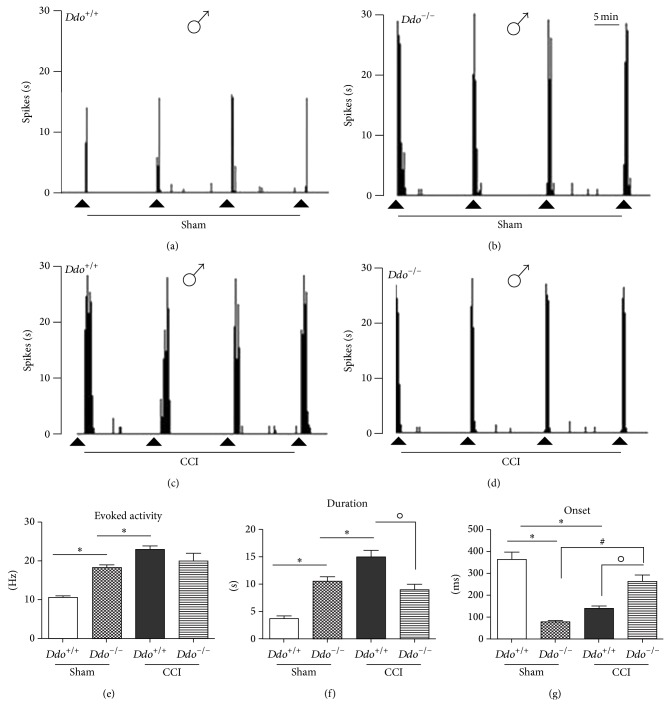
Representative ratemeters showing the responses of a single spinal NS neuron to a mechanical noxious stimulation (von Frey filaments 97.8 mN/2 sec) in* Ddo* ^+/+^ (a and c) and* Ddo* ^−/−^ (b and d) male mice 7 days after sham (a and b) or CCI (c and d) surgery. The lower panels show the evoked activity (e), the duration (f), and the onset (g) of the evoked activity induced by the noxious stimulation in NS neurons in sham and CCI* Ddo* ^+/+^ or* Ddo* ^−/−^ male mice. Small black arrows indicate the noxious stimulation on mouse hind paw and grey scale bar indicates 5 min intervals for ratemeter. Each point represents the mean ± S.E.M of 6–8 neurons of different groups of mice. *P* values <0.05 were considered statistically significant (two-way ANOVA followed by Bonferroni posttest, for comparisons between groups).  ^*^indicates significant differences versus Sham/*Ddo* ^+/+^, ^#^significant differences versus sham/*Ddo* ^−/−^, and °significant differences versus CCI/*Ddo* ^+/+^.

No change in the spontaneous firing activity of NS neurons has been found in sham wild type male mice (368 ± 28.2 ms, 10.7 ± 0.44 Hz, 3.7 ± 0.48 s, resp.; *n* = 7) (Figure 2) with respect to the naïve (not shown). Instead, we found a significant decrease in the onset and an increase in the duration and the frequency of the evoked activity of NS neurons in sham* Ddo* ^−/−^ mice (76 ± 8.3 ms, F_(3,10)_ = 98.33; 10.6 ± 0.84 s, F_(3,10)_ = 91.5; and 18.4 ± 0.63 Hz, F_(3,10)_ = 50.87, resp.; *P* < 0.05, *n* = 6) (Figure 2).

The CCI caused a significant reduction in the onset and an increase in the frequency and in the duration of the evoked activity in wild type mice (264 ± 28 ms, F_(3,10)_ = 150.99; 23 ± 0.9 Hz; F_(3,10)_ = 150 and 15.06 ± 1.23 s, F_(3,10)_ = 73.25, resp.; *P* < 0.05, *n* = 8) as compared to the sham group (368 ± 28.2 ms, 10.7 ± 0.44 Hz, 3.7 ± 0.48 s, resp., *n* = 6). Male* Ddo* ^−/−^ mice, 7 days after CCI, showed a significant increase in the onset of the evoked activity of NS neurons (264 ± 28 ms, F_(3,10)_ = 44.7, *P* < 0.05, *n* = 6) as compared to sham. A significant reduction in the duration of the evoked activity (9 ± 1 s, F_(3,10)_ = 14.33, *P* < 0.05, *n* = 6) was also observed 7 days after CCI in* Ddo* ^−/−^ male mice. No significant changes were instead observed in the frequency of the evoked activity in* Ddo* ^−/−^ male mice 7 days after the CCI (Figure 2). Representative ratemeter records show the activity of single NS neuron in wild type and* Ddo* ^−/−^ male mice 7 days after the sham or CCI surgery (Figures 2(a), 2(b), 2(c), and 2(d)).

### 3.3. Effect of the* Ddo* Gene Ablation on Mechanical Withdrawal Threshold and Thermal Withdrawal Latency

**Figure 3:**
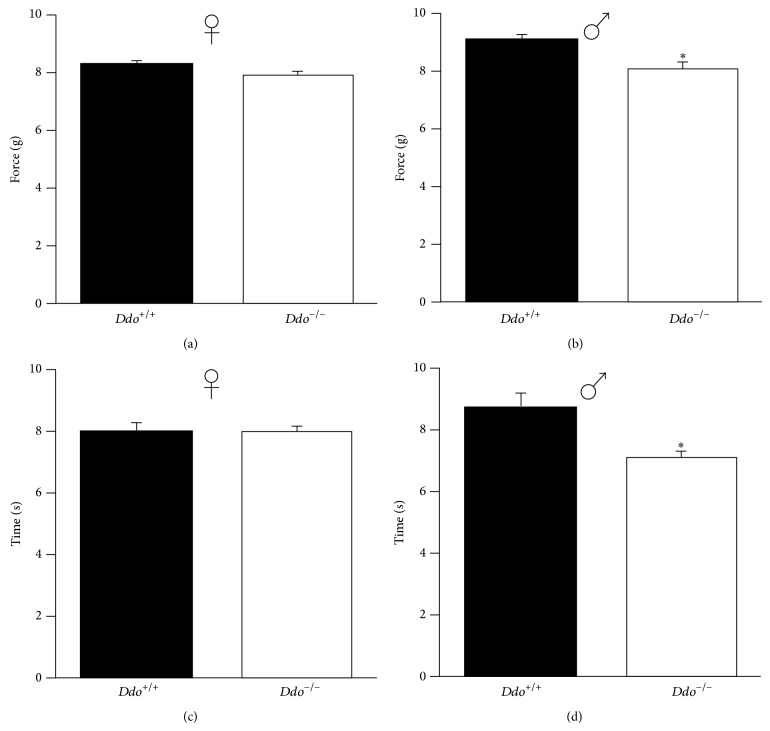
Mechanical (a and b) and thermal (c and d) nociceptive thresholds in female (♀, a and c) and male (♂, b and d)* Ddo* ^+/+^ and* Ddo* ^−/−^ naive mice. Number of mice for experimental groups: *n* = 21 (♀*Ddo* ^+/+^), *n* = 18 (♂*Ddo* ^+/+^), *n* = 23 (♀*Ddo* ^−/−^), and *n* = 15 (♂*Ddo* ^−/−^). ^*^ *P* < 0.05.

First, we have investigated whether wild type mice differ in the nociceptive mechanical and thermal threshold depending on sex. A significant difference was observed for the behavioral response to mechanical stimulus in the aesthesiometer test, with females having a lower threshold in comparison with males (*t* value: −3.542; *P* < 0.01), while no differences were detected for the thermal stimulus (*t* value: −1.161; *P* = 0.253). With regard to the deletion of the* Ddo* gene, we observed a higher sensitivity to both mechanical and thermal stimuli in male* Ddo* ^−/−^ naive mice (*t* value: −2.982; *P* < 0.05 and *t* value: −2.728; *P* < 0.01, resp.) in comparison with male wild type naive animals (Figures 3(b) and 3(d)). On the contrary, the mechanical and thermal thresholds in female* Ddo* ^−/−^ naive mice did not differ (*t* value: −1.560; *P* = 0.126 and *t* value: −0.297; *P* = 0.767, resp.) from those observed in female control naive mice (Figures 3(a) and 2(c)), suggesting that high D-Asp-mediated nociceptive response may be linked to sex-related factors.

### 3.4. Neuropathic Pain

**Figure 4:**
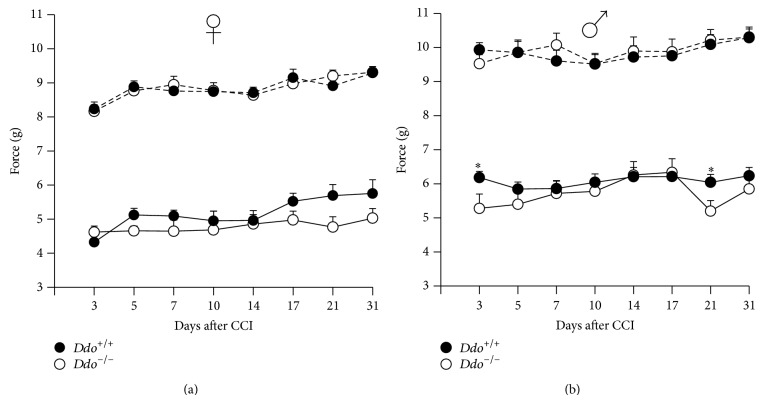
Time course of mechanical withdrawal thresholds (expressed as applied force in grams) of the ipsilateral hind paws after the chronic constriction injury (CCI) of the sciatic nerve in female (a) and male (b)* Ddo* ^+/+^ and* Ddo* ^−/−^ mice. Number of mice for experimental groups: *n* = 10 (♀*Ddo* ^+/+^), *n* = 11 (♀*Ddo* ^−/−^), *n* = 13 (♂*Ddo* ^+/+^), and *n* = 10 (♂*Ddo* ^−/−^). ^*^ *P* < 0.05.

The unilateral ligature of the sciatic nerve, performed in CCI model of neuropathic pain, induces mechanical allodynia in the hind paw ipsilaterally to the ligature. The time course of the mechanical withdrawal thresholds of both ipsilateral and contralateral hind paws, in* Ddo* ^+/+^ and* Ddo* ^−/−^ mice, has been measured for 31 days and reported in Figure 4. In all experimental groups the mechanical withdrawal thresholds decreased after CCI by about 50% in the ipsilateral, compared to contralateral hind paw. Animals withdrew their ipsilateral paw after very low stimuli (4-5 g and 5-6 g for females and males, resp.), which did not evoke reaction in contralateral paw; for this reason we considered only the response of the ipsilateral hind paw to measure mechanical allodynia. In mutant animals we observed a slight decrease in mechanical withdrawal threshold, even if two-way ANOVA for repeated measures showed a significant main effect for time (F_(7,133)_ = 4,735; *P* < 0.01 and F_(7,147)_ = 2,491; *P* < 0.01 for females and males, resp.) but not for genotype (F_(1,133)_ = 1,644; *P* = 0.215 and F_(1,147)_ = 1,362; *P* = 0.256 for females and males, resp.) or for time *x* genotype interaction (F_(7,133)_ = 1,932; *P* = 0.06 and F_(7,147)_ = 1,332; *P* = 0.238 for females and males, resp.). When a comparison between* Ddo* ^−/−^ and* Ddo* ^+/+^ mice was carried out day by day, a significant difference (*P* < 0.05) was found at day 3 and at day 21 after CCI in male mice. Indeed,* Ddo* ^−/−^ animals showed a further decrease in mechanical allodynia induced by the sciatic nerve ligation, in comparison with wild type animals.

### 3.5. Inflammatory Pain

**Figure 5:**
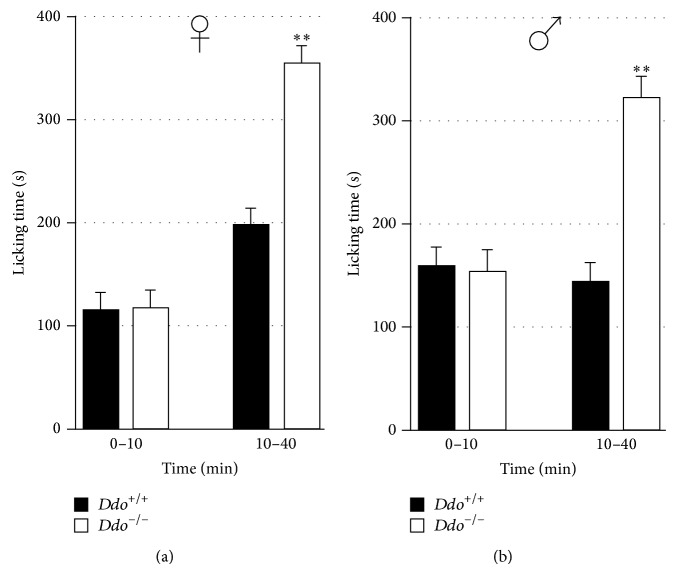
Cumulative time of the nocifensive response during phase 1 (0–10 min) and phase 2 (10–40 min) (b and d) in female (a and b) and male (c and d)* Ddo* ^+/+^ and *Ddo* ^−/−^ mice. Number of mice for experimental groups: *n* = 10 (♀*Ddo* ^+/+^), *n* = 10 (♀*Ddo* ^−/−^), *n* = 10 (♂*Ddo* ^+/+^), and *n* = 10 (♂*Ddo* ^−/−^). ^**^ *P* < 0.01.

To study behavioral response to inflammatory pain, we carried out the formalin test. Subcutaneous injection of formalin into the dorsal surface of the hind paw elicits a well-known biphasic behavioral response, as previously reported [25].  Figure 5 shows the cumulative licking time induced by formalin test during phase 1 (0–10 min) and phase 2 (10–40 min) induced by formalin. Our data indicate that, in both genders, the nocifensive responses observed during the second phase were significantly higher in* Ddo* ^−/−^ mice, when compared to wild type littermates (F_(1,18)_ = 6,590; *P* < 0.01 and F_(1,18)_ = 5,985; *P* < 0.05 for females and males, resp.). Conversely, no differences between genotypes were observed in the nocifensive responses recorded during the first phase, independently of the sex (F_(1,18)_ = 0,006; *P* = 0.938 and F_(1,18)_ = 0,010; *P* = 0.9206 for females and males, resp.).

## 4. Discussion

In the present work, we found that higher D-Asp levels in* Ddo* ^−/−^ mice reduce nociceptive threshold in physiological and chronic pain conditions. In addition, we demonstrated, by* in vivo* single unit electrophysiological recordings, that* Ddo* gene ablation consistently affects the spinal NS neuron activity in both sham and neuropathic male and female animals. Furthermore, we reported that both* Ddo* ^−/−^ female and male mutants show increased evoked activity of spinal NS neurons, as compared to wild type animals, thus suggesting that increased D-Asp levels play a role in the transmission of noxious signals in the spinal cord in physiological condition. This hyperexcitability of NS neurons in the dorsal horn of the spinal cord was also associated with a significant reduction in both mechanical and thermalnociceptive thresholds in male* Ddo* ^−/−^ mice, as compared to wild type. Interestingly, the hyperexcitability of NS neurons in the dorsal horn of the spinal cord was not associated with reduced thermal and mechanical thresholds in female* Ddo* ^−/−^ mice, highlighting a sex-dependent effect for this response. Overall, these findings indicate that high free D-Asp levels in mutant mice mediate an altered electrophysiological response and behavioral pain perception in basal conditions.

The molecular targets on which D-Asp could act are not fully known. However, NMDAR has been proposed to be involved in D-Asp-mediated central effects [26–28]. Accordingly, we have previously demonstrated that increased D-Asp levels enhance the NMDAR-dependent LTP in hippocampal slices [6–8]. Events comparable to LTP into the spinal cord have been proposed as cellular mechanisms leading to pain amplification in chronic pain conditions. Hyperalgesia and tactile allodynia are the main behavioral dysfunctions related to central sensitization associated with chronic pain [29, 30]. Consistently, NMDARs play a crucial role in the central sensitization at spinal cord dorsal horn level and NMDAR blockers have been suggested as pharmacological tools able to attenuate neuropathic pain [31, 32]. In particular, Pendersen and Gjerstad have previously shown that spinal administration of GluN2B antagonist is able to normalize the abnormal neuronal activity and attenuate the magnitude of spinal cord LTP, following peripheral nerve injury [33].

Here we have examined the effect of D-Asp on the spinal neuronal evoked activity in* Ddo* knockout mice in pathological pain condition induced by chronic constriction injury. Whilst neuropathy condition did not change or slightly reduced the nociceptive threshold in mutants, in comparison with wild type mice, paradoxically it strongly normalized the neuronal oversensitization found in naïve* Ddo* ^−/−^ mice. Indeed, in mutant mice the decrease in the onset and the increase in frequency and duration of the evoked activity to mechanical noxious stimuli of NS neurons found in sham were consistently reduced in CCI animals. Although this observation remains to be clarified at molecular level, we hypothesize the existence of NMDARs desensitization processes in mutant spinal cord under neuropathy state. In this respect, desensitization of macroscopic currents in the presence of saturating concentrations of glutamate and glycine agonists has been previously described [34] and defined as glycine-independent or glycine-insensitive. Therefore, we speculate that CCI could trigger a dramatic increase in spinal glutamate release that, combined with the high levels of D-Asp, may induce desensitization of NMDARs in* Ddo* ^−/−^ mice.

Desensitization of NMDARs in spinal cord of mutant mice could explain also behavioural data showing that neuropathic pain in* Ddo* ^−/−^ was almost unaltered compared to wild type mice except at day 3 and day 21.

Furthermore, in this study we have also examined the nocifensive behavior induced by peripheral formalin injection. The local injection of formalin into the hind paw of rodents represents a model for inducing persistent inflammatory pain, which generates a nociceptive biphasic response, oedema, and inflammation [35]. While no difference was observed in the early formalin phase, which is caused by direct activation of nociceptive sensory afferents, we found a significant increase of the late phase, which is due to the release of inflammatory mediators and also associated with central sensitization [36, 37]. The important role of D-Asp in the inflammatory component of formalin pain was put in evidence by the worsening of nocifensive response in terms of time spent in licking the injected paw in* Ddo* ^−/−^ mice, as compared with wild type animals. In the formalin pain model, the nocifensive response appears to be associated with altered spinal neuronal activity [36, 38] and thus the exacerbation of the nocifensive responses in* Ddo* ^−/−^ mice, which express NS neurons hyperactivity, and appears consistent. The disparity observed between sexes may reflect hormonal and genetic factors together with a different modulatory influence of the immune system [39, 40]. However, further studies are needed to explain the differences in pain perception between males and females and thus investigate a possible relation between* Ddo* gene deletion and gender in pain mechanisms.

## 5. Conclusions

Our study using* Ddo* ^−/−^ mice provides evidence that high levels of D-Asp affect electrophysiological and behavioral responses in physiological, inflammatory, and neuropathic pain conditions. We found an increased evoked activity of the NS neurons and a significant decrease of mechanical and thermal thresholds in* Ddo* ^−/−^ mice, as compared to controls.* Ddo* gene deletion exacerbated the nocifensive responses in the formalin test and slightly reduced pain thresholds in neuropathic mice. However, the spinal neuronal hyperexcitability caused by the* Ddo* gene deletion was no more observed 7 days after the sciatic nerve lesion. In conclusion, based on the agonistic role of D-Asp on NMDARs, the* Ddo* ^−/−^ mice may offer a substantial tool to further investigate the NMDAR-mediated mechanisms involved in neuropathic pain.
